# Estimation of Excess Deaths Associated With the COVID-19 Pandemic in Istanbul, Turkey

**DOI:** 10.3389/fpubh.2022.888123

**Published:** 2022-07-25

**Authors:** Abdullah Ucar, Seyma Arslan

**Affiliations:** ^1^Anafartalar Primary Healthcare Center, Ministry of Health, Istanbul, Turkey; ^2^Arnavutköy District Health Directorate, Ministry of Health, Istanbul, Turkey

**Keywords:** COVID-19, excess mortality, excess deaths, expected deaths, *P*-Score, Istanbul, Turkey, TURCOVID19

## Abstract

**Background and Objectives:**

The official number of daily cases and deaths are the most prominent indicators used to plan actions against the COVID-19 pandemic but are insufficient to see the real impact. Official numbers vary due to testing policy, reporting methods, etc. Therefore, critical interventions are likely to lose their effectiveness and better-standardized indicators like excess deaths/mortality are needed. In this study, excess deaths in Istanbul were examined and a web-based monitor was developed.

**Methods:**

Daily all-cause deaths data between January 1, 2015- November 11, 2021 in Istanbul is used to estimate the excess deaths. Compared to the pre-pandemic period, the % increase in the number of deaths was calculated as the ratio of excess deaths to expected deaths (*P*-Scores). The ratio of excess deaths to official figures (T) was also examined.

**Results:**

The total number of official and excess deaths in Istanbul are 24.218 and 37.514, respectively. The ratio of excess deaths to official deaths is 1.55. During the first three death waves, maximum *P*-Scores were 71.8, 129.0, and 116.3% respectively.

**Conclusion:**

Excess mortality in Istanbul is close to the peak scores in Europe. 38.47% of total excess deaths could be considered as underreported or indirect deaths. To re-optimize the non-pharmaceutical interventions there is a need to monitor the real impact beyond the official figures. In this study, such a monitoring tool was created for Istanbul. The excess deaths are more reliable than official figures and it can be used as a gold standard to estimate the impact more precisely.

## Introduction

Since 31 December 2019, the number of confirmed cases and deaths due to COVID-19 around the world has reached 255 million and 5 million, respectively. The number of cases and deaths in Turkey has reached 8 million and 70.000, respectively (1). The WHO has published daily epidemiologic data between 21 January- 16 August 2020, and weekly thereafter. With a dashboard created by Johns Hopkins University on February 22, 2020, global data was visualized in real-time and a pandemic monitor was created (2, 3). In Turkey, the number of cases and deaths is announced on the official website of the Ministry of Health (MoH) daily.

To assess the impact of the pandemic, the numbers of daily cases and deaths are the main indicators that affect all others (4). Regular access to accurate information on these indicators from a reliable source is critical in pandemic governance. Planning and implementation of non-pharmaceutical interventions (NPIs) and interpretation of results are assessed *via* these reference indicators. In this context, the accurate detection of the number of cases and deaths is critical to a precision assessment of the real impact in the field and for the re-optimization of the NPIs.

Certain factors (testing policies, case definitions, reporting procedures, etc.,) may differ from country to country. This makes it difficult both to evaluate the number of cases and deaths and to make comparisons between countries (5). In this case, better-standardized indicators and criteria are required to assess the impact accurately and to minimize the difficulties on country comparisons. At this point, the epidemiological concept “excess mortality/death” is used as a better-standardized indicator than official COVID-19 death numbers (6).

Excess mortality refers to the total number of direct and indirect deaths during a public health crisis that occurs in greater numbers than expected in the pre-crisis period (4, 7). The number of deaths in public health crises is higher than in the pre-crisis period and deaths may result from both the causes directly related to the crisis or issues that are secondary to the crisis (8). Direct deaths during the COVID-19 pandemic are deaths directly related to the COVID-19 infections. Secondary deaths are caused by not being able to access health services due to the blocked health system, lockdown, fear of disease, etc., (9). Suicides due to stress caused by the pandemic may also constitute a part of indirect deaths (10). Excess mortality is recommended as the gold standard for a more accurate estimation of the number of deaths caused by COVID-19 and for the real impact of the pandemic (6, 11–13).

In the first months of the pandemic, the total number of monthly deaths in the United States was higher than expected based on the average number of deaths between 2014 to 2019 (14). The comparison of the number of deaths in 2020–2021 to the period 2016–2019 shows the first peak in excess mortality occurred in March-April 2020 in various European countries. After a few months of decline, a new increase started in August 2020, reaching a peak in November 2020 (14). These developments have shown the need for the creation of regularly updated dynamic datasets and digital platforms where these datasets are analyzed in real-time to monitor the trend of excess mortality over time, to compare countries, and to assess the impact of interventions. Several platforms have been collecting data on excess deaths from around the world, of which EuroMOMO, Eurostat, UNDATA, SMTF, mortality.org, and the World Mortality Dataset Project are the leading ones. Based on these datasets, excess mortality data has been visualized in real-time on interactive web-based platforms. Our World in Data, The Economist, the Financial Times, and the New York Times are prominent among these platforms (8).

Unfortunately, Turkey is not among the countries evaluated on these platforms and research (15, 16). The main reason is that there is no dynamic and open access dataset containing the daily number of deaths for Turkey. However, the daily numbers of all-cause deaths in Istanbul are presented as open data on the state's official website, turkiye.gov.tr. Istanbul includes 18.66% of Turkey's population and is one of the 12 NUTS-1 regions in Turkey. Istanbul is the most crowded city in Turkey and has a cosmopolitan population from all over the country, so the statistics for Istanbul should give a good idea of the general population.

Most of the excess mortality studies in Turkey were conducted in the early stages of the pandemic. These cross-sectional studies are static, as they do not offer a constantly updated dataset or a dynamic visualization. This study aims to examine the number of excess deaths, the ratio of excess deaths to official deaths, and the size of the death waves during the COVID-19 pandemic in Istanbul. A dynamic dataset generated by sarkac.org and TURCOVID19 Project in Turkey was used in this study and presented as open access data (Supplementary Material 1). Additionally, an interactive web-based dashboard was created that was updated in real-time depending on the dataset.

## Materials and Methods

### Study Design

In this study, the number of excess deaths during the COVID-19 pandemic has been examined using the daily all-cause death statistics in Istanbul between January 1, 2015 and November 11, 2021. This study consists of 4 stages. In the first stage, the average number of daily deaths over the pre-pandemic 5 years (2015–2019) was taken as the baseline and the daily number of excess deaths in 2020 and 2021 was calculated. In the second stage, to enable comparisons with other countries and regions we calculated the measure (*P*-Score) suggested in the Aron and Muellbauer 2020 study (17). In the third stage, the size and duration of the death waves in the pandemic period were examined. In the fourth stage, the official COVID-19 deaths and the excess deaths in Istanbul were compared, and an examination of the extent to which the pandemic's true impact went beyond the official statistics was conducted. The calculations were made available to the public through a dynamic open dataset and an interactive web-based excess death monitor was formed.

### Data Source, Scope, and Preparation

Daily all-cause death data from Istanbul Metropolitan Municipality was publicly available on the state's official website, turkiye.gov.tr. The daily all-cause death data include given names, surnames, and the total number. With the cooperation of sarkac.org and the TURCOVID19 project, daily all-cause death numbers of Istanbul since 2015 have been collected, and a dynamic dataset that is regularly updated has been created.

The dataset includes 365 days of the year as rows, and all-cause death numbers for the years 2015–2021 as columns. In the dynamic dataset, the minimum, maximum, average, and median values of the pre-pandemic years' data are calculated automatically for each day.

### Inclusion and Exclusion Criteria

Data from February 29 are excluded due to the associated confounding effect in the calculations because the date February 29 only occurs once every 4 years in the Gregorian calendar.

### Data Analysis

#### First Stage: Calculating the Excess Deaths for Istanbul

The average values of the daily all-cause deaths in Istanbul for the pre-pandemic period (2015–2019) were calculated for each day. Although the simple average method has some limitations in estimating expected deaths based on pre-pandemic data, the simple mean method was preferred because comparative studies showing that estimations based on Poisson, Gamma or Binomial distributions are superior in this regard are still limited. number of average daily deaths in pre-pandemic and post pandemic years has been compared with the Mann-Whitney *U*-test.

For the day t, the average daily number of all-cause deaths in pre-pandemic period (expected) were subtracted from the number of daily all-cause deaths during the pandemic period (observed). In the calculation, the five-year average number of all-cause deaths for each day of the year expresses the expected death value for that day (D_expected_), and the number of daily all-cause deaths during the pandemic period expresses the observed death value (D_observed_). In this case, the equation for the number of excess deaths on a day t (Dt_excess_) is:


$$\begin{array}{l} Dt_{\text{excess}}=Dt_{\text{observed}}-Dt_{\text{expected}} \end{array}$$

#### Second Stage: Establishing *P*-Score

The ratio of excess deaths to expected deaths presented as *P*-Scores. *P*-Score shows the percentage difference how much the number of excess deaths during the pandemic period deviated from the expected number of deaths. The equation for the *P*-Score on the day t (Dt) is given by:


$$\begin{array}{l} \text{P}-Score_{\text{t}}=\frac{Dt\;\left(excess\right)}{Dt\;\left(expected\right)} \end{array}$$

#### Third Stage: Analysis of Death Waves During the Pandemic Period

The parts of the daily death charts where the number of observed deaths increased and peaked over the pandemic period were considered as “death waves”. The total number of deaths during these waves and the maximum values in each wave were calculated and compared.

#### Fourth Stage: Official Death Numbers and Comparison With Excess Deaths

The estimated excess deaths were compared to the official number of COVID-19 deaths announced by the Turkish Ministry of Health (MoH). The daily official death numbers due to COVID-19 in Turkey have been announced as the total daily value for the country. Between 28 June 2020 and 25 October 2020, the number of official deaths at the NUTS-1 level was published as weekly reports by the MoH. Istanbul is one of Turkey's twelve NUTS-1 regions on its own. For this reason, the official death numbers in Istanbul were recorded weekly between 28 June and 25 October 2020 and made available as open data on the TURCOVID19 website (Supplementary Material 2). According to the first official report (June 28, 2020) the ratio of the total official COVID-19 deaths registered in Istanbul to the official number of deaths registered in the whole country was %52,72. This ratio decreased over time and fell to 33.2% in the latest published report (October 25, 2020). Namely, at the relevant date 33.2% of official deaths due to COVID-19 in Turkey occurred in Istanbul. After this date, the publication of the reports was stopped the death numbers continued to be announced for Turkey in general. This ratio was used as the reference parameter to estimate the number of daily official deaths in Istanbul. In this calculation, the official number of deaths in Istanbul (Ot_Istanbul_) was estimated by multiplying the total number of deaths in Turkey on the day t (Ot_Turkey_) by 0.332. In addition, calculations for the period (27 June—25 October 2020) of the official reports were made separately, with and without extrapolation.


$$\begin{array}{l} Ot_{\text{istanbul}}=Ot_{\text{Turkey}}\;\times \;0.332 \end{array}$$

In addition, total excess deaths in Istanbul ($\overset{t}{\underset{i=1}{\sum }}Dt_{excess}$) were compared to total official deaths ($\overset{t}{\underset{i=1}{\sum }}Ot_{istanbul}$) to determine the extent to which the pandemic's true impact exceeded the official figures. The ratio of these two parameters (T) has been calculated as follows:


$$\begin{array}{l} T=\;\overset{t}{\underset{i=1}{\sum }}Dt_{excess}÷\;\overset{t}{\underset{i=1}{\sum }}Ot_{istanbul} \end{array}$$

## Results

**Figure 1:**
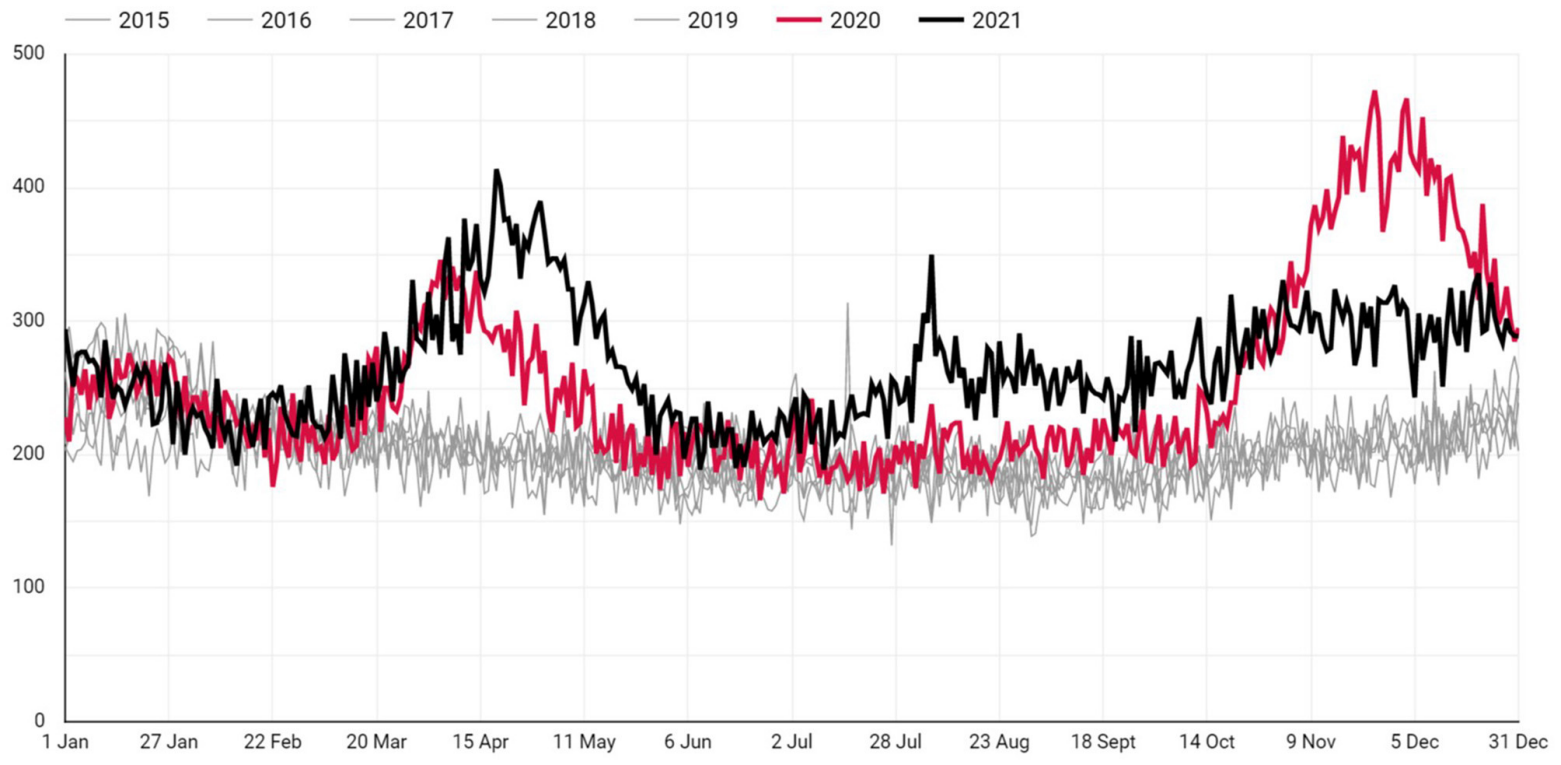
The daily number of all cause deaths in Istanbul (2015–2021).

The daily number of deaths in 2020 and 2021 in Istanbul comparison to previous years are as illustrated in Figure 1. The total number of excess deaths in Istanbul up to the date November 11, 2021 is 37.514. Daily number of deaths during pre-pandemic and post-pandemic years are shown in Table 1. The daily average of excess deaths since the beginning of 2020 has been 55.17 (SD: 57.94 Max: 264.8). The number of daily average deaths in post-pandemic years is significantly greater than in the pre-pandemic years (*p* < 0.001).

**Table 1:** The average number of daily deaths in Istanbul during pre-pandemic^*^ and post-pandemic^**^ years.

**Years**	***N***	**Minimum**	**Maximum**	**Mean**	**Std. deviation**
*2015	365	148	243	193.33	21.86
*2016	365	147	314	202.20	25.71
*2017	365	139	299	205.65	28.07
*2018	365	155	263	204.37	20.09
*2019	365	132	306	208.53	28.44
**2020	**365**	**166**	**473**	**253.65**	**68.63**
**2021	**365**	**189**	**414**	**266.97**	**43.09**

*Bold values represent the numbers during the pandemic period*.

**Figure 2:**
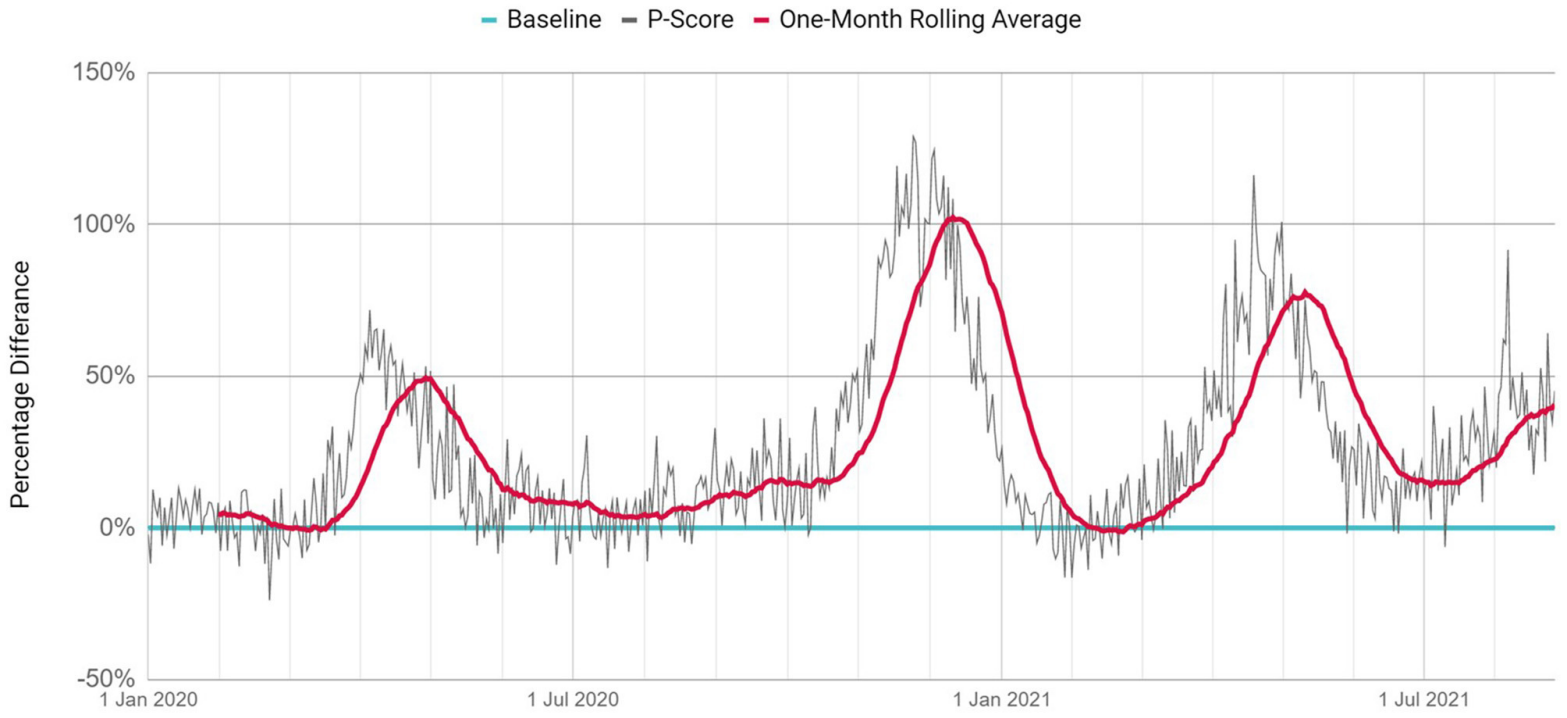
The ratio of excess deaths to expected deaths (*P*-Scores).

*P*-Scores from the beginning of 2020 are shown in Figure 2. On a daily and monthly basis respectively, *P*-Scores have reached a peak of 71.8 and 49.5% during the first wave, 129.0 and 102.32% during the second wave, 116.3 and 77.61% during the third wave.

The first wave occurred between March and May 2020, the second wave occurred between October and January 2020, and the third wave occurred between February and June 2021. The excess deaths in these three waves are 4.604, 11.934, and 9.221, respectively. The largest number of excess deaths occurred in the second wave. The magnitude of the second wave was 2.59 times greater than that of the first wave and 1.29 times greater than that of the third wave. The third wave was the first wave of 2021. During this wave, the number of excess deaths between February and June 2021 (*n* = 9.221) was more than double that of the same period during the previous year (*n* = 4.604).

In the first wave of 2020, the number of excess deaths (*n* = 4.604) during March-May was 3.06 times that of the official number of deaths (*n* = 1.507). The daily maximum number of excess deaths and the official deaths were 144 and 42, respectively. In this period, one out of every three deaths was recorded in the official records. In the four-month period between June and September 2020, the first wave faded because of the measures taken, and the gap between official deaths and excess deaths decreased. During this period, the number of excess deaths (*n* = 2.027) was 1.67 times that of the official number of deaths (*n* = 1.213).

The second wave of 2020 lasted for 4 months, from October 2020 to January 2021. In this wave, the number of excess deaths (*n* = 11.934) was double that of the official death count (*n* = 5.945). The daily maximum number of excess deaths and the official deaths were 264 and 86, respectively. For the first time in this wave, on 31 December 2020, the number of excess deaths fell below the official number of deaths and decreased rapidly, in a trend that continued for 3 months.

The third wave lasted for 5 months, from February to June 2021. During this wave, the number of excess deaths (*n* = 9.221) was 1.16 times that of the official number of deaths (*n* = 7.908). The daily maximum number of excess deaths and the official deaths were 223 and 131, respectively. For the first time in this wave, on 12 March 2021, the number of excess deaths increased rapidly, exceeding the official number of deaths, and continuing in this way for 2 months. After official and excess death numbers had progressed at the same rate for 1 month following May 23, with the commencement of the normalization phase in Turkey the number of excess deaths began to rise again after 23 June. At the time of this study, the fourth death wave was on the rise.

The number of total estimated official deaths ($\overset{t}{\underset{i=1}{\sum }}Ot_{istanbul}$) and excess deaths ($\overset{t}{\underset{i=1}{\sum }}Dt_{excess}$) in Istanbul since the beginning of the pandemic have been 24.218 and 37.514, respectively. The total number of excess deaths is 1.55 (T) times that of the official number of deaths.

**Figure 3:**
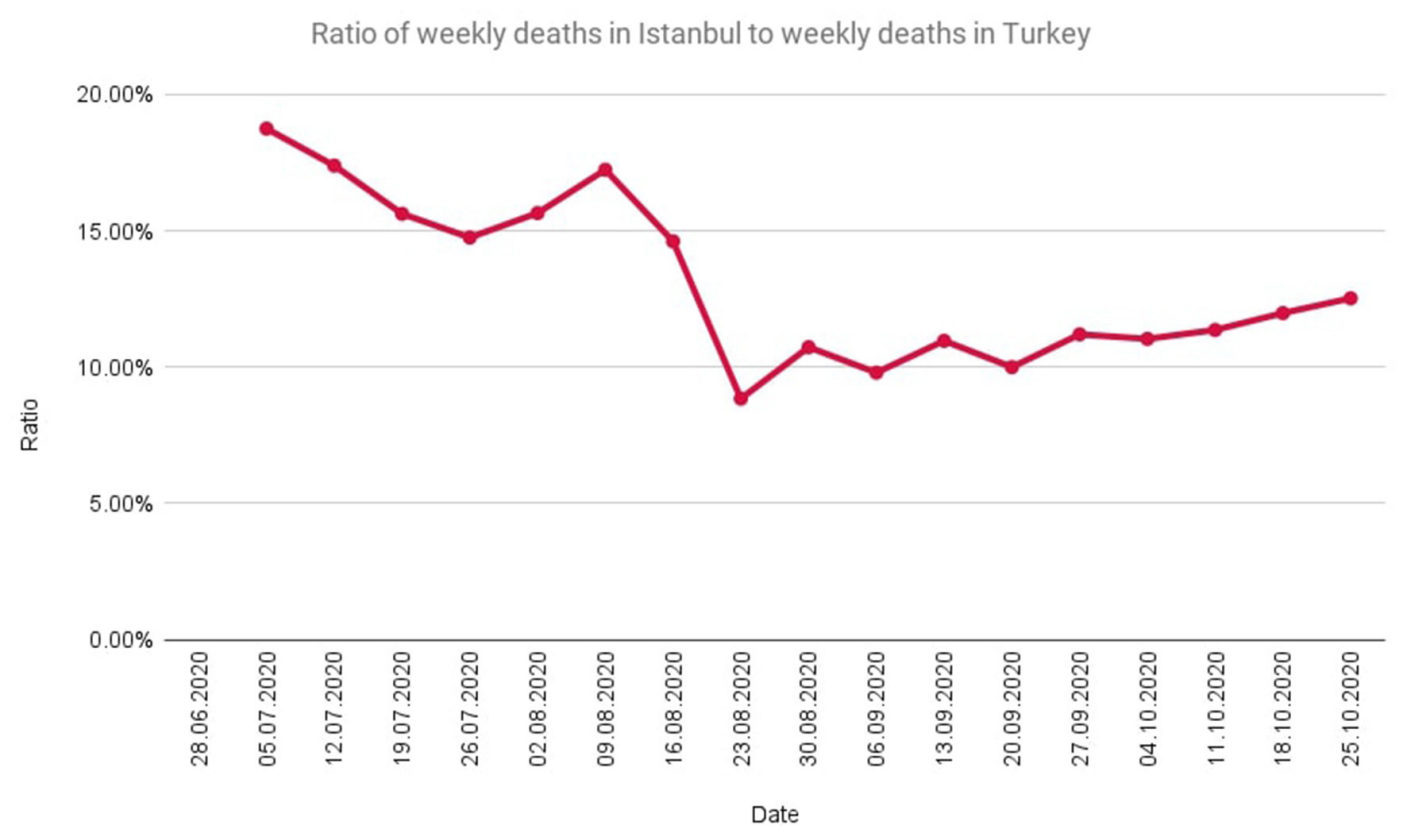
The ratio of weekly deaths in Istanbul to weekly deaths in Turkey during the time period in which official reports are published.

According to the calculations made for the period in which the official reports are published (27 June−25 October 2020), the extrapolated T parameter is 1.57, while it is 4.34 according to the non-extrapolated calculations. In this period, the ratio of the weekly official death toll in Istanbul to the overall death toll in Turkey was as in Figure 3. The official number of COVID 19 deaths in Istanbul during this period was 12.04% of the overall number in Turkey during this period.

## Discussion

The number of deaths in Istanbul during the COVID-19 pandemic was considerably higher than in the pre-pandemic period. While the number of daily deaths between 2015–2019 was within a certain range, it increased in 2020 and 2021, creating large waves of deaths. Death waves last between 3 and 5 months on average. These waves tend to fall when non-pharmaceutical interventions are applied and rise during periods of relaxation.

**Figure 4:**
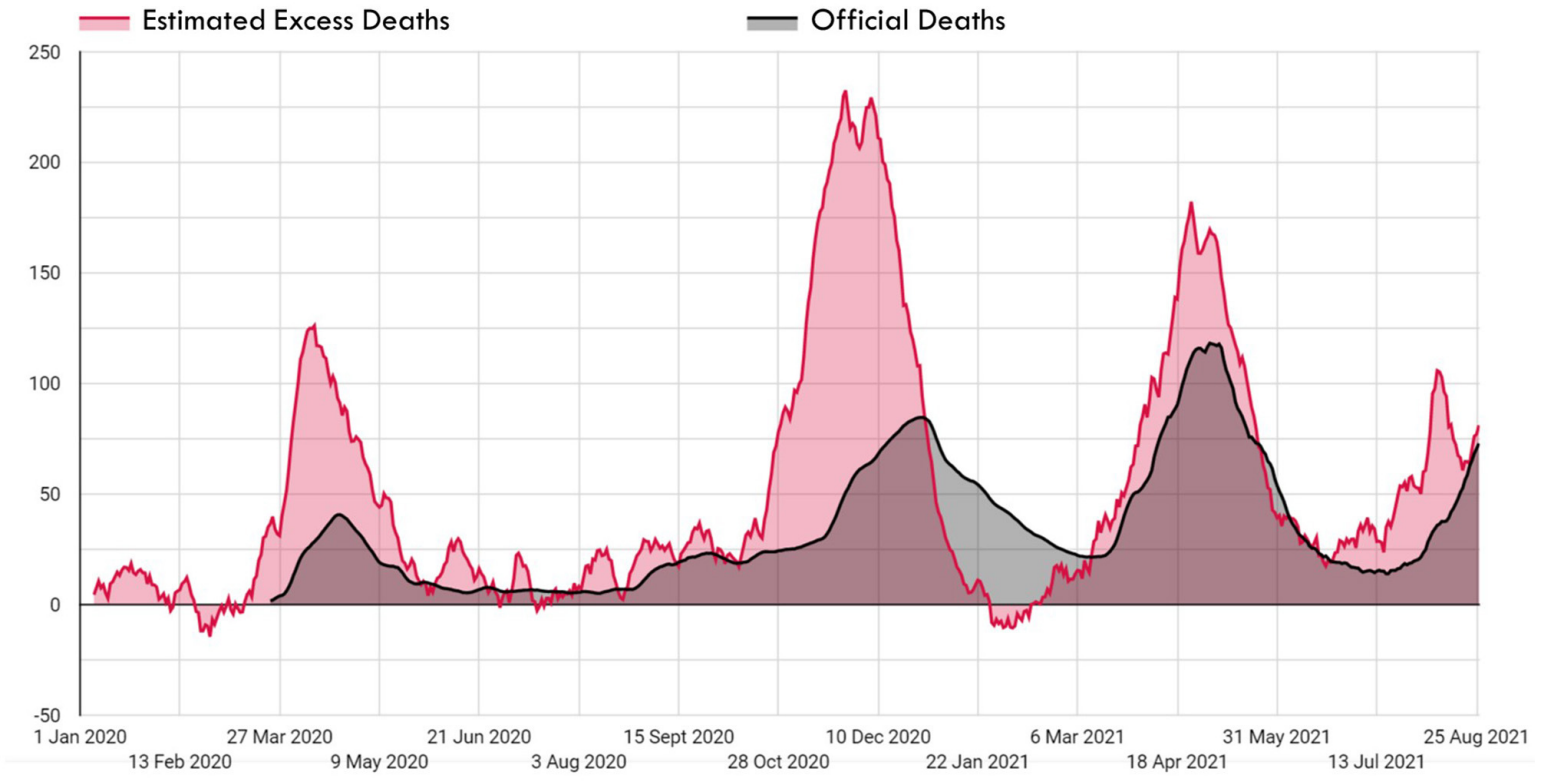
Excess deaths and official deaths during the COVID-19 pandemic in Istanbul.

The first confirmed COVID-19 case and death in Turkey were announced on 11 and 17 March 2020, respectively. However, as shown in Figure 4, excess deaths in Istanbul started to increase earlier, in the first days of March 2020. This indicates that there were cases of COVID-19 in Istanbul long before the first case was announced.

The ratio of excess deaths to expected deaths (*P*-Score) and the ratio of excess deaths to official deaths (T) have been used as indicators throughout the pandemic. In Istanbul, the maximum values of monthly-based *P*-Scores were realized in the first wave as 49.5%, in the second wave as 102.3 and the third wave as 77.6%. In a study that included 94 countries around the world, the top three countries with the highest *P*-Scores between January and June 2020 were Peru (146%), Ecuador (77%), and Bolivia (61%) (8). In Europe during the first wave the highest excess deaths were observed in Spain (80.8%), Belgium (73.1%), and Netherland (53.8%); in the second wave, Poland (97.0%), Bulgaria (94.4%), and Slovenia (93.2%); and in the third wave, Bulgaria (76.9%), Poland (65.3%), and Czechia (62.0%). Compared to European countries, the total number of deaths per unit population in Turkey seems to be lower, but in terms of excess deaths, it is seen that the excess deaths in Istanbul are close to the peak values in Europe.

The ratio of the total number of excess deaths to official deaths (T) shows the underreporting level of the COVID-19 deaths and this value is 1.55 for Istanbul. Additionally, this ratio shows that official deaths can just explain 61.52% of the excess deaths in Istanbul. The remaining 38.47% include undetected COVID-19 deaths and indirect deaths. However, the T value also varies seasonally in the same country. T value was 3.06 in the first wave in Istanbul and decreased to 1.16 in the third wave. As well as the calculations without extrapolation using direct official figures over the time period of official reports show that the T value was 4.34. This high rate confirms that the excess deaths in the relevant time period far outnumber the official deaths. In Italy, Spain, the Netherlands, and the United Kingdom, the T value was at a maximum of ~1.5 in the first wave but dropped to ~1.0 in the second wave. The T value was below 3 for most countries in the world, but much higher values were observed in some countries. The highest values were 100 in Tajikistan, 51 in Nicaragua, 31 in Uzbekistan, 14 in Belarus, and 13 in Egypt (8).

The use of official death numbers to measure the effectiveness of responses to the pandemic or to make comparisons between countries raises several issues. Official numbers are affected by many factors. For example, the low number of cases and deaths related to COVID-19 in some countries may be due to the low number of tests (18, 19). In addition, definitions of COVID-19 cases and deaths may be different between countries. Some countries include only deaths of PCR-positive people in their COVID-19 deaths, while others also include suspected COVID-19 deaths (8). Another confounder is that deaths that occurred in hospitals do not represent all COVID-19 deaths (6). In this context, the Statistics Netherlands's (CBS) study proposes that all excess deaths be considered COVID-19 deaths (20). And a study supports this proposal by expressing excess mortality offers a much more robust assessment opportunity compared to the official number of COVID-19 deaths (14).

Due to the measures taken against the pandemic, there may be indirect deaths that have been prevented. These prevented deaths can result in a shift in the number of deaths due to various causes. According to our findings, the daily death numbers in January and February in 2021 vary more and are distributed in a wider range than the other months of the year during 2015–2019. The number of excess deaths decreases to negative values in January-February. It is thought that this variability may be due to influenza-related deaths experienced in the winter season. In theory, unless there are other confounding factors, the number of excess deaths should not be less than the official COVID-19 deaths. The paradox here is thought to be caused by a confounder. The most likely reason for this is the decrease in influenza deaths in the winter period due to pandemic measures, which were replaced by COVID-19 deaths. According to the studies, there was a decrease in deaths from other infectious diseases, particularly seasonal flu, as a result of social interventions (8, 13, 14, 18–24). In the United States, the 99,3% decrease in influenza cases in the 2020–2021 winter season compared to 2019–2020 supports the shift argument presented above (19). Another confounder may be early deaths. According to Institute for Health Metrics and Evaluation (IHME), deaths from chronic diseases of the heart and respiratory system decreased by 2% in some European countries in the middle of the year 2020. Large numbers of elderly and chronically ill people are likely to have died early in the year as a result of COVID-19, and their early deaths may explain the negative additional deaths during the winter period (19). However, other effects reduce deaths in the pandemic. According to IHME, mobility restrictions reduced the number of traffic accident-related deaths by 5%, and 215.000 traffic accident-related deaths were prevented globally in 2020 (19). In this regard, during the pandemic, it is possible the effects that increase and decrease the number of excess deaths balance each other.

## Conclusion

In this study, the number of excess deaths which is one of the most important indicators for monitoring the real impact of the pandemic has been estimated for Istanbul. These estimations give an idea about the excess deaths around Turkey. The outcomes of this study support that the progression of the pandemic should be monitored not just with official COVID-19 deaths, but also utilizing excess deaths. The number of excess deaths begins to rise earlier than the number of official deaths. Delays are inevitable in social intervention measures which are decided based on the official deaths. The excess deaths have predictive value for official death numbers. However, there are also periods when the number of excess deaths falls below the official numbers, or even becomes negative. In light of the literature, we also address the possible causes of this condition in our study.

Excess mortality is a key indicator for monitoring the true consequences of the pandemic, developing a more precise pandemic management strategy, ensuring that interventions are implemented without delay and on time, and, indeed, increasing the effectiveness of such interventions. To be faster than the spread of the pandemic is a key factor in governing it. At this point, the application of digital technology to this field opens up new frontiers for us.

Previous studies on excess death in Turkey are at the cross-sectional level, whereas evolving digital technologies make it possible to build up-to-date monitoring screens based on dynamic datasets, allowing decision-makers and scientists to monitor the pandemic situation in real-time (25, 26). In this regard, digital epidemiology applications have a significant role in monitoring the pandemic's consequences.

## Limitations of the Study

In this study to estimate the expected deaths as a baseline in the pandemic period, the simple average method is used with 2015–2019 data. Although this method has some limitations it is used in the Eurostat study (14).

The use of the simple average method when calculating the expected number of deaths according to the pre-pandemic period may cause the baseline to be underestimated for countries where the number of deaths increased by years, or to be overestimated for those with decreased death numbers. This baseline method doesn't consider changes in the population's age structure and reduction in mortality rates over years.

The multiplier (0.332) used to estimate how many of the COVID-19 deaths in Turkey occurred in Istanbul includes the possibility of extrapolation in the calculation of future data after the latest report released by MoH. This multiplier is based on the latest report of MoH and there is no other official reference to use in Turkey.

Excess mortality estimates assume that daily deaths in the pre-pandemic years occurred under ordinary conditions. There was no extraordinary public health crisis in Turkey in the pre-pandemic years 2015–2019.

There is also the possibility of displacing deaths caused by the pandemic with deaths prevented due to preventive measures against the pandemic in excess mortality estimates. In this study, the subject is discussed based on the example of the displacement of influenza deaths and COVID-19 deaths during post-pandemic years.

## Data Availability Statement

The datasets presented in this study can be found in online repositories. The names of the repository/repositories and accession number(s) can be found in the article/Supplementary Material.

## Author Contributions

Conceived and designed the analysis, contributed data or analysis tools, and wrote the paper: AU and SA. Collected the data and performed the analysis: AU. All authors contributed to the article and approved the submitted version.

## Conflict of Interest

The authors declare that the research was conducted in the absence of any commercial or financial relationships that could be construed as a potential conflict of interest.

## Publisher's Note

All claims expressed in this article are solely those of the authors and do not necessarily represent those of their affiliated organizations, or those of the publisher, the editors and the reviewers. Any product that may be evaluated in this article, or claim that may be made by its manufacturer, is not guaranteed or endorsed by the publisher.

## Supplementary Material

Supplementary Material 1 Dynamic dataset of daily official and excess deaths in Istanbul.

Supplementary Material 2 Weekly death numbers at NUTS-1 level in Turkey.
